# Cross-level transformation of creativity from entrepreneurs to organizations

**DOI:** 10.3389/fpsyg.2024.1278996

**Published:** 2024-03-08

**Authors:** Xiu Yang, Meng Zhang, Zixuan Meng, Yingxian Shen, Xiaomin Du

**Affiliations:** ^1^Accounting and Auditing College, Guangxi University of Finance and Economics, Nanning, China; ^2^Business School, Shandong Normal University, Jinan, China; ^3^Management Science Department, City University of Hong Kong, Hong Kong, Hong Kong SAR, China; ^4^Management School, Hainan University, Haikou, China; ^5^International Business School, Hainan University, Haikou, China

**Keywords:** entrepreneurial individual creativity, organizational creativity, platform leadership, organizational culture, cross-level transformation of creativity

## Abstract

With the intensification of competition in the business environment, organizational creativity is increasingly becoming crucial for organizations to build competitive advantages and promote organizational development. For innovative enterprises, their entrepreneurs largely determine the development orientation of the enterprise. They are one of the most critical factors determining the level of corporate innovation, but there need to be more effective creativity transformation path to pursue innovation development. The findings in this study show that entrepreneurial individual creativity has a significant positive effect on organizational creativity, platform leadership mediates the path of creativity transformation across hierarchical levels, and organizational culture has positive moderating effect between platform leadership and organizational creativity. The study results explain the transformation mechanism of creativity from the entrepreneur's perspective, expand the potential transformation path of organizational creativity, and are instructive for enhancing organizational creativity.

## 1 Introduction

In an innovation-driven society, creativity is increasingly becoming the critical element in driving organizational development (Makri and Scandura, 2010; Woods et al., 2018). More and more organizations seek to create unique competitive advantages through innovation to survive and thrive in the competitive business environment (Parjanen, 2012; Blomberg et al., 2017). Most innovative companies are still in the early stage of development and need more significant certainty and clarity. The level of organizational creativity largely determines the development prospects of innovative companies (Chang and Chen, 2020). Entrepreneurial individual creativity is the source of innovation, and organizational creativity is the key to maintaining innovation drive and competitive advantage, which together determine the level of innovation of the firm (Pirola-Merlo and Mann, 2004; Yeh-Yun Lin and Liu, 2012). Therefore, it is important to explore the transformation path from entrepreneurial individual creativity to organizational creativity to develop innovative industries (Chang and Chen, 2020).

However, there are still three areas for improvement in the current research on organizational creativity. First, existing research is more concerned with the transformation path of creativity from employees to the organization, but recruiting only creative employees does not meet the organization's need in terms of innovation (Blomberg et al., 2017). And entrepreneurs are employees with special status and positions, with far more power and influence than ordinary employees (Hughes et al., 2018). Still, only some have explored the extent to which the individual creativity traits of entrepreneurs affect the level of innovation in the company, which is not conducive to the further improvement of the level of organizational creativity (Moultrie and Young, 2009; Gao et al., 2020). Second, scholars have explored the mechanisms of their effects on creativity in terms of leadership types, such as Lutz Allen et al. (2013) and Herrmann and Felfe (2014), who explored the relationship between transformational as well as aissez-faire leadership and organizational creativity (Lutz Allen et al., 2013; Herrmann and Felfe, 2014). However, platform leadership still lacks an effective transformation path in the process of transformation from individual creativity to organizational creativity. There needs to be research to confirm whether platform leadership can carry the transformation between the two, and this research situation is not conducive to further expanding the potential paths and transformation mechanisms of creativity from the perspective of leadership types. Finally, organizational culture is usually studied as a driver of organizational creativity. Still, existing studies paid less attention to whether organizational culture interacts with other drivers in the transformation path of creativity, and the role of organizational culture in the path from leadership type to organizational creativity still needs to be further studied (Chitsazan et al., 2017).

To address the research gaps mentioned above, the main tasks of this study are as follows: First, due to the special status and influence of entrepreneurs, this paper explored the mechanism of their roles in the transformation path of organizational creativity from the perspective of entrepreneurial individual creativity, and confirmed that they are the direct drivers of organizational creativity and can motivate entrepreneurs to adopt a platform leadership thus further contributing to the enhancement of organizational creativity. Secondly, platform leadership emphasizes equality and shared relationships among members of the organization, which is conducive to employees following the leader to achieve the company's objective and enhance the organization's creativity. Moreover, because of its role as a link between the upper and lower levels of the organization, platform leadership can take over the transformation of entrepreneurial individual creativity to organizational creativity, i.e., platform leadership plays a mediating role in the transformation path of creativity. Finally, this paper reveals the mechanism between platform leadership, organizational culture and organizational creativity, and explores the moderating role of organizational culture. In transforming from platform leadership to organizational creativity, organizational culture plays a positive moderating role, leading to the further improvement of organizational creativity. The theoretical foundations of this study include Social Cognitive Theory and Social Information Processing Theory. Social Cognitive Theory emphasizes that behavior can shape the environment and that individuals can adjust their attitudes and behaviors based on signals released in the environment. Namely, individuals are not only the shaper of organizational environment, but also the product of organizational environment at the same time (Schunk and DiBenedetto, 2020). And Social Information Processing Theory highlights how individuals adjust themselves to be congruent with the environment based on the information they gather from it. The prerequisite of Social Information Processing Theory is that individual would adjust their behavior and attitude based on social environment, which means we can predict and manage the behavior of organizational member based on organizational environment (Meyer, 1994).

This study aims to explore the pathways of creativity transformation through a survey conducted among members of innovative Chinese enterprises. By investigating this specific organizational context, the research seeks to gain valuable insights into the process of converting creativity into innovation. This study established and improved a cross-level transformation model of organizational creativity from an entrepreneurial perspective, explored entrepreneurial individual creativity as an antecedent factor, and confirmed the positive moderating role of organizational culture in the transformation path of platform leadership and organizational culture. Through the questionnaire survey administered to organizational members and entrepreneur, this study aims to examine the factors and mechanisms that facilitate or hinder the transformation of creative ideas into tangible outcomes within Chinese companies. By identifying the unique pathways and challenges in this context, this research can contribute to a better understanding of the dynamics of creativity transformation and inform strategies to promote innovation in Chinese enterprises.

## 2 Research framework and hypothesis

**Figure 1:**
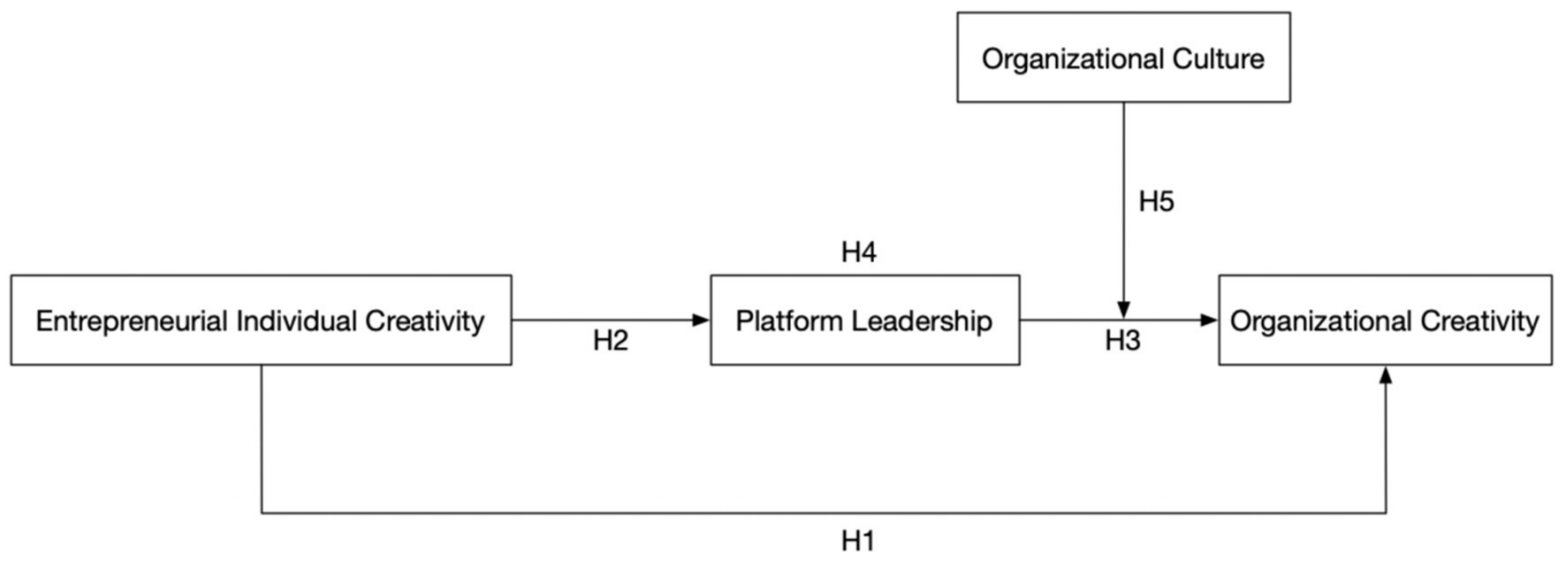
Cross-level creativity transformation model from entrepreneurs to organizations.

According to existing researches, the level of organizational creativity depends on entrepreneurial individual creativity, platform leadership, and organizational culture (Andleeb et al., 2019; Schunk and DiBenedetto, 2020; Yang et al., 2022). Based on Social Cognitive Theory and Social Information Processing Theory, this paper established a cross-level creativity transformation model from entrepreneurs to organizations, this study's theoretical model and hypotheses are shown in Figure 1.

Creativity is a kind of intangible asset without specific measurements, which is the source of organizational innovation and the key to winning in competition (Baron and Tang, 2011; Andleeb et al., 2019). For innovative firms, it is even more important to rely on creativity to attract potential customers, (Ramos et al., 2022) and thus increase the market share of the firm to win in the business competition (Pagano et al., 2018). Entrepreneurial individual creativity refers to the ability of entrepreneurs to produce new ideas of practical significance through appropriate information and knowledge (Zampetakis and Moustakis, 2006). According to the Social Cognitive Theory, individuals will pursue innovative entrepreneurial goals if they believe their creativity can achieve the desired results (Schunk and DiBenedetto, 2020). According to the Social Information Processing Theory, entrepreneurs, as leaders of innovative enterprises, are important sources of information in the organization, able to provide a variety of information and resources to organizational members, and able to influence the attitudes and behaviors of organizational members (Meyer, 1994). As entrepreneurs are different from ordinary employees in terms of influence and power, their creativity largely affects the overall level of creativity of the organization, the higher the individual creativity of entrepreneurs, the higher the organizational creativity and innovation potential that they can unleash (Yeh-Yun Lin and Liu, 2012; Xu and Zhao, 2020). Therefore, this paper proposes the following hypotheses:

**H1:** Entrepreneurial individual creativity is positively correlated with organizational creativity.

Platform leadership is grounded on the construction of digital information platform, highlighting the importance of entrepreneurs dynamically stimulating the inherent qualities of their employees through their inclusive attributes and personal charisma in a top-down fashion (Yang et al., 2022). Platform leadershipaims to foster change-oriented behaviors, ultimately leading to the collective development of employees, entrepreneurs, and the organization (Yang et al., 2022). According to Social Information Processing Theory, entrepreneurs are important sources of information in organizational contexts and can provide various information resources within the organization (Xu and Zhao, 2020). Since the risks faced in the process of innovation and entrepreneurship are unknown, the material resources, information resources , and organizational support provided by the organization can help organizational members to reduce their fear and aversion to risk (Catalano et al., 2018). To allow organizational members to give full play to their creativity, and to form the organizational atmosphere of proactive change within the organization, entrepreneurs tend to send signals of innovation to organizational members utilizing the digital information platform. These platforms have powerful data integration capabilities, which can positively influence the performance of creativity within the organization (Benitez et al., 2022). By leveraging these platforms, entrepreneurs can send signals of innovation to organizational members, encouraging them to embrace and contribute to a culture of creativity and change, which will help them to form a benign atmosphere of innovation (Yang et al., 2022). Therefore, this paper proposes the hypothesis:

**H2:** Entrepreneurial individual creativity is positively correlated with platform leadership.

Organizational creativity refers to the innovative perspectives implemented to achieve the enhancement of production, processes, and services, which can improve organizational performance and increase the core competitiveness of the enterprise to achieve substantial development of the enterprise (OUARI and Lefkir, 2022). Existing studies have shown that, at the organizational level, leadership type is an important factor influencing organizational innovation behavior (Khedhaouria et al., 2015). While the platform leadership emphasizes the equal and shared relationship between organizational members, it can adopt a bottom-top approach to lead employees, respond to changes in the external environment of the organization, and ultimately promote the achievement of organizational goals (Morris et al., 2005). Organizations adopting platform leadership can establish an information platform that integrates resources from all parties, shapes the organizational learning atmosphere, and thus motivates organizational members to share information, which is conducive to the implementation of innovation at the organizational level (Yang et al., 2022). Therefore, this paper proposes the hypothesis:

**H3:** Platform leadership is positively correlated with organizational creativity.

Entrepreneurial individual creativity is the ability of entrepreneurs to find new opportunities, or to transform ideas into actual organizational outputs (Fillis and Rentschler, 2010). At present, there is a lack of effective transformation mechanism between entrepreneurial individual creativity and organizational creativity, so this paper introduces platform leadership to explore its role in the transformation path from entrepreneurial creativity to organizational creativity. With the development of information network technology, the external environment faced by innovation-oriented enterprises has become more unpredictable, and to seek survival and development in this environment, entrepreneurs expect organizations to show a trend of decentralization and de-leadership (Vaara et al., 2021). Platform leadership focuses on building a digital information platform within the organization, which is characterized by inclusiveness, personal charisma, platform building, change planning, platform optimization, and common growth, and can achieve the mutual development of employees, entrepreneur, and organization, as well as integrating more high-quality resources to enhance the level of creativity of the entire organization (Yang et al., 2022). According to the Social Exchange Theory, the two parties who establish a social exchange relationship follow the principle of reciprocity. If the organization can give enough authorization, resources, and platform to its members, in exchange, the employees will also enhance their sense of responsibility and belonging to the organization, which will stimulate the initiative of the organization members to innovate, and further enhance the organization's overall level of creativity (Fuller et al., 2006). Platform leadership emphasizes support, tolerance, and understanding of organizational members, and when organizational members feel organizational support, it can reduce their negative emotions such as fear and dissatisfaction about the risks involved in innovation work (Eisenberger et al., 2020). According to the Social Information Processing Theory, within an organization, information affects the behavior of organizational members by influencing individual perceptions (Meyer, 1994). Therefore, platform leadership can enhance the power of entrepreneurial individual creativity , which is conducive to the development of innovation within the organization, and the further enhancement of the organization's overall level of creativity. Therefore, this paper proposes the hypothesis:

**H4:** Platform leadership mediates between entrepreneurial individual creativity and organizational creativity.

Organizational culture is a kind of umbrella term for beliefs about shared values that can shape behavioral norms within an organization and create an organizational way of life (Al Shehri et al., 2017). Organizational culture can play a supportive, interactive, sharing role within the organization, which can motivate organizational members to think creatively and be oriented to work creatively, a strong organizational culture is the key to motivating organizational members to think and act creatively (Andleeb et al., 2019). If the organization expects to be able to form an the innovative atmosphere within the organization, it is necessary to make its organizational culture sufficiently flexible to provide the organization members with the opportunity to think and behave creatively. If the organization expects to form an innovative atmosphere within the organization, it is necessary to make its organizational culture flexible enough to provide a comfortable and flexible working environment and atmosphere for the members, to enhance their sense of responsibility and sense of belonging to the organization (De Clercq and Mustafa, 2023), which is conducive to the development of innovative work within the organization, and thus enhance the level of organizational creativity for the whole (Jeong et al., 2017). According to Bandura's Reciprocal Determinism, organizational culture is one of the key factors driving the innovative and entrepreneurial behaviors of organizational members (Zhao et al., 2020). Under the influence of the robust organizational culture, entities that embrace platform leadership can exhibit heightened inclusiveness in navigating the challenges associated with innovation and entrepreneurship (De Clercq and Mustafa, 2023). This fosters an environment conducive to alleviating employees' feelings of insecurity and fear associated with the risks posed by innovation. Guided by platform leadership, the organization becomes more dedicated to the strategic planning and execution of innovative initiatives, thereby bolstering the overall level of organizational creativity (Carmeli et al., 2014). Therefore, this paper proposes the hypothesis:

**H5:** Organizational culture positively regulates the relationship between platform leadership and organizational creativity.

## 3 Materials and methods

### 3.1 Data collection

In the competitive business environment, creativity is the key for enterprises, especially innovative enterprises, aiming to build core competitiveness and realize their survival and development. Therefore, in this paper, entrepreneurs and organization members of innovative enterprises were selected as survey respondents, encompassing different developing levels of enterprises located in representative cities in the north, middle, and southeast of China. And online questionnaires were generated using the Questionnaire Star platform, corresponding links and QR codes of the questionnaires were sent through social platforms, such as WeChat and QQ, to invite the respondents to access the questionnaires. The questionnaire collection lasted ten months from January 2023 to November 2023, and 458 valid samples were obtained. The descriptive statistics of the samples are shown in Table 1.

**Table 1:** Characteristics of research sample (N = 458).

**Control variables**	**Item**	**Frequence**	**Percentage**	**Control variables**	**Item**	**Frequence**	**Percentage**
Gender	Male	239	52.18	Industry	Advertising	57	12.45
	Female	219	47.82		Design	85	18.56
Age	<=30	122	26.64		Software	65	14.19
	30–35	94	20.52		Filming and TV	53	11.57
	36–40	91	19.87		Music and painting	51	11.14
	41–45	76	16.59		Publishing and performing arts	47	10.26
	>=46	75	16.38		Others	100	21.83
Educational background	High school	80	17.47	Years of establishment	<=1	71	15.50
	Bachelor	138	30.13		1–3	80	17.47
	Master and Doctor	126	27.51		3–5	94	20.52
	Others	114	24.89		5–10	111	24.24
Working Seniority	<=2	110	24.02		>=10	102	22.27
	2–4	106	23.14	Firmsize	<=100	81	17.69
	5–8	85	18.56		101–200	109	23.80
	9–15	66	14.41		201–300	98	21.40
	>=16	91	19.87		301–500	67	14.63
					>500	103	22.49

### 3.2 Variable measurement

In order to guarantee the reliability and validity of the measurement scales, the items of the mature measurement scales were selected for this study and refined with the context and characteristics of the study. The scales in this paper are all scored on Likert 5-point scale, with 1–5 indicating from fully inconsistent to fully consistent.

1. Entrepreneurial individual creativity.
According to Gao et al. (2021), entrepreneurial individual creativity contains five items (Gao et al., 2021).
2. Platform leadership.
Drawing on the scale of Yang et al. (2022) and other scholars, platform leadership is measured in six dimensions: tolerance, charisma, platform building, revolution planning, platform optimization, and mutual growth (Yang et al., 2022). In total, this measure contains 25 items..
3. Organizational creativity.
Drawing on the scale of Gao et al. (2021) to measure organizational creativity, the measure contains a total of five items (Gao et al., 2021).
4. Organizational culture.
Based on Anne Kennan et al. (2006), this study measured organizational culture in five dimensions, development of employees, interpersonal harmony, customer orientation, social responsibility, and dare to innovate (Anne Kennan et al., 2006; Tsui et al., 2006). Organizational culture contains a total of 23 items.

### 3.3 Reliability and validity tests

Descriptive statistics and correlation coefficients are shown in Table 2. The results show that there is a positive correlation between entrepreneurial individual creativity and platform leadership (cor = 0.195, p <0.01), a positive correlation between entrepreneurial individual creativity and organizational creativity (cor = 0.228, p <0.01), and a positive correlation between platform leadership and organizational creativity (cor = 0.302, p <0.01), and that the above results initially validate the hypotheses H1, H2, and H3.

**Table 2:** Descriptive statistics results with correlation coefficients.

**Variable**	**Average**	**Standard deviation**	**1**	**2**	**3**	**4**
1 Entrepreneurial individual creativity	3.697	1.024	1			
2 Platform leadership	3.620	0.718	0.195^**^	1	
3 Organizational culture	3.661	0.681	0.228^**^	0.302^**^	1	
4 Organizational creativity	3.722	0.838	0.458^**^	0.411^**^	0.211^**^	1

^**^p <0.01(two-tailed test).

According to Table 3, the Cronbach's α values for the four variables were >0.9, making them suitable for further confirmatory factor analysis.

**Table 3:** Reliability Test Results.

**Variables**	**Items**	**Cronbach** α	**CR**
Entrepreneurial Individual Creativity	5	0.920	0.925
Platform Leadership	25	0.957	0.957
Organizational Culture	23	0.949	0.949
Organizational Creativity	4	0.842	0.851

As shown in Table 4, the confirmatory factor analysis was conducted using SPSS 27.0 for scales of this study and the validity test was performed directly. The results showed that the standardized load factors were all within the acceptable range (>0.600), which indicates that there is a strong correlation between the latent variables and the analytic item measures. The value of CR>0.900, indicating high convergent validity, and KMO>0.70, which indicates that the validity of the study data is feasible.

**Table 4:** Scale items and validity tests.

**Factor (latent variable)**	**Measurement items (significan variables)**	**Standard load factor**
**Entrepreneurial individual creativity (KMO=0.885, CR=0.925)**		
EIC1	I usually search out new creative elements and inspiration, and then utilize those ideas in my creative business.	0.861
EIC2	I am not afraid to take risks.	0.903
EIC3	I usually suggest new ways to achieve goals and objectives.	0.899
EIC4	I often have a fresh approach to problems.	0.907
EIC5	In general, I am a good source of creative ideas.	0.620
**Platform leadership (KMO=0.955, CR=0.957)**		
Tolerance		
PL1	My leader does not mind if his subordinates are better than himself in some aspects.	0.616
PL2	My leader does not mind occasional mistakes in his subordinates' work.	0.622
PL3	My leader does not mind sharing honors and opportunities with his subordinates.	0.610
PL4	My leader does not mind and often encourages his subordinates to give him advice.	0.668
PL5	My leader respects his subordinates' differences in personalities and abilities.	0.654
Charisma		
PL6	My leader always stays positive in good times and bad.	0.629
PL7	My leader can put himself in his subordinates' shoes.	0.679
PL8	My leader does not give up when things get tough.	0.686
PL9	My leader can make decisions quickly and accurately when encountering emergencies or important cases.	0.625
PL10	My leader can deal with problems objectively and fairly.	0.650
Platform Building		
PL11	My leader has full confidence in his subordinates' work ability and personal character.	0.646
PL12	My leader believes that the interests of his subordinates agree with those of the organization.	0.718
PL13	My leader is committed to continuous improvement of existing organizational systems.	0.709
PL14	My leader has sufficient socio-economic resources to help the organization achieve its goals.	0.748
Revolution Planning		
PL15	My leader has a long-term plan for developing the company or team.	0.717
PL16	My leader can quickly identify and summarize the essence of problems.	0.741
PL17	My leader can clearly set and describe the vision of the organization.	0.746
Platform Optimization		
PL18	My leader is good at motivating subordinates to pursue higher goals.	0.700
PL19	My leader encourages subordinates to embrace and learn all the knowledge beneficial to organizational development and personal improvement.	0.734
PL20	My leader encourages subordinates to constantly seek new ideas and approaches in solving problems.	0.729
PL21	My leader communicates frequently and proactively with subordinates emotionally.	0.747
Mutual Growth		
PL22	My leader often pays attention to their growth and gives his subordinates guidance and education.	0.740
PL23	My leader continues to learn advanced professional knowledge and leadership skills.	0.626
PL24	My leader creates opportunities to fully empower subordinates to take charge of a project.	0.797
PL25	My leader often communicates with subordinates about new technologies and knowledge to help them grow.	0.603
**Organizational culture (KMO=0.947, CR=0.949)**		
Development of employees		
OCU1	My organization pays attention to the personal development of employees.	0.672
OCU2	My organization develops the potential of employees.	0.677
OCU3	My organization trusts its employees.	0.711
OCU4	My organization values employee opinions.	0.666
OCU5	My organization provides knowledge and skills training.	0.634
InterpersonalI Harmony		
OCU6	My organization attaches great importance to team building.	0.694
OCU7	My organization supports the spirit of cooperation.	0.673
OCU8	My organization promotes feelings/sharing among employees.	0.709
OCU9	My organization encourages cooperation.	0.692
OCU10	My organization's employees care about each other.	0.693
Customer orientation		
OCU11	My organization can meet the needs of customers to the maximum extent.	0.621
OCU12	My organization emphasizes customer benefits.	0.628
OCU13	My organization can provide first-class service.	0.635
OCU14	My organization believes that customers come first.	0.664
OCU15	My organization provides sincere service.	0.669
Social responsibility		
OCU16	My organization demonstrates social responsibility.	0.697
OCU17	My organization has a sense of mission to serve the society.	0.708
OCU18	My organization emphasizes social benefits as well as economic benefits.	0.698
OCU19	My organization promotes the development of society.	0.718
Dare to innovate		
OCU20	My organization is open to change.	0.655
OCU21	My organization can continuously develop new products and services.	0.642
OCU22	My organization encourages innovation.	0.629
OCU23	My organization boldly adopted high technology.	0.620
**Organizational creativity (KMO=0.784, CR=0.851)**		
OCR1	The work (including business ideas, products, services) my firm produces is creative.	0.625
OCR2	The work (including business ideas, products, services) my firm produces is novel and original.	0.855
OCR3	The work (including business ideas, products, services) my firm produces is characteristic.	0.885
OCR4	The work (including business ideas, products, services) my firm produces satisfies market demands in creative industry.	0.683

## 4 Empirical testing and analysis

### 4.1 Research method selection

The purpose of this study is to explore the cross-level transformation path and mechanism from entrepreneurial individual creativity to organizational creativity. According to the existing research, this paper explores the transformation of organizational creativity from entrepreneurial individual creativity, platform leadership and organizational culture. This paper adopts multilevel regression analysis to analyze the correlation between variables, the core of multilevel regression analysis is still regression analysis, but it can be divided into multiple layers, which is conducive to exploring whether the newly put variables have explanatory strength for the model. In this paper, the PROCESS tool is used to test the mediating effect and moderating effect, and the Bootstrap method is adopted to sample the mediating effect, and an estimated confidence interval is constructed by sampling with put-back, which is a more efficient method and has no restriction on the distribution of mediating samples.

### 4.2 Correlation test

In this paper, hypotheses were tested using multilevel regression using SPSS 27.0 software. As shown in Table 5, the multilevel regression analysis for organizational creativity involves a total of three models. The explained variable of the model is organizational creativity, and the independent variables of model 1 are control variables (gender, age, educational background, working seniority, industry, years of establishment, and firmsize), and model 1 examines the effects of the control variables. Model 2 adds entrepreneurial individual creativity on the basis of model 1, and the results show that there is a significant positive correlation between entrepreneurial individual creativity and organizational creativity (β = 0.376, *p* < 0.001), and H1 is proved. Model 3 adds platform leadership on the basis of model 2, and the results show that there is a significant positive correlation between platform leadership and organizational creativity (β=0.387, *p* < 0.001), and H3 is proved.

**Table 5:** Correlation results of multilevel rgression tests.

**Category**	**Variable**	**Organizational creativity**			**Platform leadership**	
		**Model 1**	**Model 2**	**Model 3**	**Model 4**	**Model 5**
Control variables	Gender	-0.004	-0.022	-0.029	0.026	0.019
	Age	0.072^**^	0.065^**^	0.063^**^	0.008	0.005
	Educational background	-0.051	-0.034	-0.039	0.006	0.013
	Working seniority	-0.005	-0.017	-0.006	-0.022	-0.027
	Industry	-0.009	-0.013	-0.002	-0.029	-0.030
	Years of establishment	-0.002	-0.031	-0.009	-0.046	-0.057^*^
	Firmsize	0.012	0.012	0.004	0.019	0.019
Explanatory variables	Entrepreneurial individual creativity		0.376^**^	0.319^**^		0.148^**^
	Platform Leadership			0.387^**^		
	R^2^	0.020	0.228	0.331	0.018	0.062
	Adjusted R^2^	0.005	0.214	0.318	0.002	0.045
	F Value	1.300	16.562^***^	24.654^***^	1.160	3.679^***^

^*^p < 0.05, ^**^p < 0.01, ^***^p < 0.001.

As shown in Table 5, the multilevel regression analysis for platform leadership involves two models. Among them, the independent variables in Model 4 are control variables (gender, age, educational background, working seniority, industry, years of establishment, firmsize), and Model 5 adds entrepreneurial individual creativity on the basis of Model 4. The explained variable of this model is platform leadership, and the results show that there is a significant positive correlation between entrepreneurial individual creativity and platform leadership (β= 0.148, *p* < 0.01), and H2 is proved.

### 4.3 Mediation effect test

This paper was validated using the PROCESS tool in SPSS 27.0. The results are shown in Table 6, under the measurement of Bootstrap method with 5,000 times of repeated sampling, the coefficient of entrepreneurial individual creativity to platform leadership is 0.1374, and the coefficient of platform leadership to organizational creativity is 0.3895 in 95% confidence range, after calculating 0.1379*0.3892 ≈ 0.0537, which is consistent with the results of the analysis results, and together with the confidence interval (LLCI=0.0256, ULCI=0.0846) does not contain 0, which means that it is proved that platform leadership can act as a mediating variable and play a significant mediating effect between entrepreneurial individual creativity and organizational creativity, and H4 is confirmed.

**Table 6:** Summary of intermediating role test results.

**Items**	**Path coefficients**	**Standard deviation**	***P*** **-value**	**LLCI**	**ULCI**
EIC → PL → OCR	0.3747	0.0341	0.0000	0.3078	0.4417
EIC → PL	0.1379	0.0322	0.0000	0.0746	0.2011
PL → OCR	0.3892	0.0461	0.0000	0.2985	0.4789
EIC → OCR	0.3211	0.0323	0.0000	0.2575	0.3846
EIC → OCR (Control of PL)	0.0537	0.0150	0.0000	0.0255	0.0850

### 4.4 Moderation effect test

In this paper, the multilevel regression in SPSS 27.0 was used to conduct the effect test with moderated mediating utility, and the control variables were tested together, and the results are shown in Table 7, the effect value of the interaction term (platform leadership × organizational culture) is 0.108, and the p-value is 0.047 which is less than 0.05, indicating that organizational culture has a significant effect on the relationship between in platform leadership and organizational creativity is significant.Therefore, organizational culture can play a positive moderating role between platform leadership and organizational creativity, H5 has been proved.

**Table 7:** Multilevel Regression result of moderation effect test.

**Category**	**Variable**	**Organizational creativity**		
		**Model 1**	**Model 2**	**Model 3**
Control variables	Gender	-0.004	-0.018	-0.024
	Age	0.072^**^	0.067^**^	0.064^**^
	Educational background	-0.051	-0.055	-0.053
	Working seniority	-0.005	0.007	0.010
	Industry	-0.009	0.005	0.005
	Years of establishment	-0.002	0.019	0.020
	Firmsize	0.012	0.001	0.000
Explanatory variables	Platform leadership		0.451^**^	0.058
	Organizational culture		0.113^**^	-0.285
	Platform leadership × Organizational Culture			0.108^*^
	R^2^	0.020	0.196	0.203
	Adjusted R^2^	0.005	0.180	0.185
	*F* value	1.300	12.136^***^	11.391^***^

^*^p < 0.05, ^**^p <0.01, ^***^p <0.001.

## 5 Discussion

This study established a cross-level creativity transformation model from entrepreneurs to organizations based on social cognitive theory and social information processing theory. Overall, the cross-level creativity transformation model expands the potential paths of creativity transformation and improves the research on platform leadership and organizational culture in the mechanism of creativity transformation. This study presents a robust predictive ability for organizational creativity, which can well reflect the formation causes and transformation paths of organizational creativity. The research findings of this study are threefold:

First, this paper validated the positive impact of entrepreneurial individual creativity on organizational creativity. Individual creativity is the source of innovation, however, exploring the transformational path of organizational creativity only from the perspective of employees cannot meet the needs of organizational innovation (Blomberg et al., 2017). Existing research suggests that entrepreneurs are superior to ordinary employees in terms of power, status, and influence (Hughes et al., 2018), therefore, entrepreneurial individual creativity is an important driver of organizational creativity, and can positively influence the enhancement of organizational creativity (Yeh-Yun Lin and Liu, 2012; Xu and Zhao, 2020). This paper confirms the cross-level transformation path of creativity from entrepreneurs to organizations through empirical tests, verifies the important role of entrepreneurial individual creativity in the transformation of creativity, indicating its contribution to the further enhancement of organizational creativity.

Second, this study explored the mechanism of leadership type from the perspective of creativity transformation. Existing studies mostly focus on the relationship between leadership type and organizational creativity (Herrmann and Felfe, 2014; Sirkwoo, 2015), but lack research on whether it can undertake the transformation of creativity from individuals to organizations across hierarchical levels (Ozsahin and Sudak, 2015). Drawing on the social information processing theory and the social exchange theory, platform leadership can transfer information and resources across hierarchical levels by building a digital information platform (Yang et al., 2022). Platform leadership owns the nature of equality and sharing, which can establish the innovative working atmosphere within organization, allowing organization members to give full play to their creativity (Benitez et al., 2022). Constructing digital platofrm helps individuals to carry out the innovative and creative work at the organizational level, and enhances the innovative atmosphere of the whole organization (Xu and Zhao, 2020; Yang et al., 2022). On the basis of the above research, this paper confirms that platform leadership can undertake the transformation of creativity between entrepreneurs and organizations, therefore, entrepreneurs with platform leadership, specifically in entrepreneurial enterprise, are able to utilize digital platform to assist the enhancing for the level of organizational creativity.

Finally, this paper explored the interacting role of organizational culture in the path of creativity transformation. Organizational culture is the important motivator of innovative behavior (Zhao et al., 2020). The emotional value provided by organizational culture to organizatinal members can enhance their emotional commitment to organization (Tsui et al., 2006), showing a stronger sense of responsibility and belonging, and driving them to carry out innovative and entrepreneurial behaviors (Jeong et al., 2017). This paper explained the deepening role of organizational culture for platform leadership, explored the transformational path of organizational creativity in the perspective of organizational culture, and confirmed the effectiveness of the path through empirical data. Namely, this study validated the significant positive moderating effect of organizational culture, which is of strong practical significance for enhancing the level of organizational innovation from the level of organizational culture.

In summary, this study expanded the existing research about organizational creativity from several perspectives and confirmed the validation in the context of entrepreneurial enterprise in China. Particularly, a cross-level creativity transformation model was established from entrepreneurs to organizations, confirming the validity of the creativity transformation path from entrepreneurs to organizations. Furthermore, this study supplemented the gaps of the existing creativity research in the platform leadership perspective, expanded the transformation path of organizational creativity, improved the application space of the existing theories to a certain extent, which has practical significance for further enhancement of the level of organizational creativity from the entrepreneurial perspective , specifically in the context of China. From a practical perspective, entrepreneurs can implement a series of measures to enhance the creativity of their organizations. This includes supporting the construction of a digital information platform, promoting the engagement in creative activities, and integrating elements of Chinese traditional culture to create a more syncretic organizational culture aligned with the context of China. The cross-level creativity model developed in this study identifies the transformation path of creativity from entrepreneurs to the organization. This model proves valuable in effectively harnessing the positive impact of platform leadership and organizational culture, enhancing creative activities and thinking. It provides a practical methodology for organizations to establish a creative environment, offering concrete steps for entrepreneurs to foster innovation within their organizations. In future research, it would be beneficial to explore universal transformational approaches that can be applied to enhance organizational creativity, which can be universally applied across different organizational contexts to foster and improve creativity.

## Data availability statement

The original contributions presented in the study are included in the article/supplementary material, further inquiries can be directed to the corresponding author.

## Ethics statement

Ethical approval was not required for the studies involving humans in accordance with local legal and institutional requirements. The studies were conducted in accordance with the local legislation and institutional requirements. The participants provided their written informed consent to participate in this study.

## Author contributions

XY: Funding acquisition, Writing—original draft, Writing—review & editing. MZ: Investigation, Funding acquisition, Writing— original draft. ZM: Investigation, Writing—original draft, Data curation, Methodology, Software, Writing—review & editing. YS: Data curation, Writing—original draft. XD: Writing—review & editing, Methodology, Formal analysis.
